# Edição de Outubro de 2024, vol. 121(10): e20240190

**DOI:** 10.36660/abc.20250038

**Published:** 2025-02-05

No Artigo de Revisão “Anormalidades Cardíacas nas Síndromes Hipereosinofílicas”, com número de DOI: https://doi.org/10.36660/abc.20240190, publicado no periódico Arquivos Brasileiros de Cardiologia, Arq Bras Cardiol. 2024; 121(10):e20240190, na página 1, no resumo, alterar a frase: “O envolvimento cardíaco na SHE ocorre em três fases: necrótica inicial, trombótica e necrótica final” por: “O envolvimento cardíaco na SHE ocorre em três fases: inflamatória, trombótica e fibrótica.”

