# Supplementary material for: Edição de Outubro de 2024, vol. 121(10): e20240190
Source: Arq Bras Cardiol. 2025 Feb 5;122(2):e20250038. [Article in Portuguese] doi: 10.36660/abc.20250038 (PMC11805570; doi:10.36660/abc.20250038)
Supplement: Translation

# Translation: October 2024 Issue, vol. 121(10): e20240190

**DOI:** 10.36660/abc.20250038i

In the Review Article “Cardiac Abnormalities in Hypereosinophilic Syndromes”, with DOI: https://doi.org/10.36660/abc.20240190i, published in the journal Arquivos Brasileiros de Cardiologia, Arq Bras Cardiol. 2024; 121(10):e20240190, on page 1, change the sentence in the abstract: “The cardiac involvement of HES occurs in three phases: initial necrotic, thrombotic, and finally necrotic.” to: “The cardiac involvement in HES occurs in three phases: inflammatory, thrombotic, and fibrotic.”
